# Assessing the feasibility of partner-implemented digital payment systems for health workers: stakeholder perspectives from Uganda’s yellow fever mass vaccination campaign – a qualitative study

**DOI:** 10.1136/bmjgh-2024-017472

**Published:** 2025-09-18

**Authors:** Michael Ediau, Elizabeth Ekirapa Kiracho, Juliet Aweko, Charles Opio, Maggie Ssekitto Ashaba, Peter Waiswa

**Affiliations:** 1 Department of Health Policy, Planning and Management, Makerere University School of Public Health, Kampala, Uganda; 2 Building Research Initiatives Advancing Global Health Equity (BRIDGE), San Diego State University School of Public Health, San Diego, California, USA; 3 Center of Excellence for Maternal, Newborn and Child Health, Makerere University School of Public Health, Kampala, Uganda; 4 Global Public Health, Karolinska Institute, Stockholm, Sweden; 5 Busoga Health Forum, Jinja, Uganda

**Keywords:** Health services research, Vaccines, Prevention strategies, Qualitative study

## Abstract

**Background:**

The heightening of efforts to eradicate vaccine-preventable diseases through mass vaccination campaigns has contributed to a growing demand for effective and efficient payment mechanisms for frontline vaccination workers in large-scale campaigns. Subsequently, the Uganda Ministry of Health (MoH) adopted digital payments for campaign workers, which were either implemented by the government or partners. We specifically explored stakeholder perspectives on the feasibility of partner-implemented digital payment to front-line health workers in phase one of the yellow fever mass vaccination campaign in Uganda.

**Methods:**

We employed a cross-sectional qualitative study. The study area comprised four districts (Amuru, Lira, Hoima and Kikuuba) purposively selected from 51 phase one yellow fever vaccination campaign districts. We collected data through 37 qualitative interviews (25 key informant interviews (KIIs) and 12 in-depth interviews (IDIs)). IDI participants included vaccination health workers (n=12). KII interview participants included district technical officials (n=14), representatives of digital payment implementing partners at district and national levels (n=7) and MoH staff (n=4). All interviews were audio-recorded and later transcribed and analysed using thematic analysis.

**Results:**

The partner-implemented payment system was linked with perceived improvements in timeliness and the increased likelihood of beneficiaries receiving complete payment. Despite these benefits, some payment delays were reported. These delays were mainly attributed to incomplete and inaccurate participant payment information. Health workers said they were more motivated because they felt assured of being paid and receiving the full amount.

**Conclusions:**

Despite drawbacks, partner-led digital payment of health workers was perceived as a feasible strategy promoting timely, complete payments. Relevant stakeholders should ensure accurate, complete and timely capture and verification of health workers’ payment details to counter payment delays. We recommend more rigorous evaluations to determine whether a partner-implementation of digital payment is more effective than government-led payment in different settings.

**WHAT IS ALREADY KNOWN ON THIS TOPIC**

- Few studies have evaluated the adoption of digital payment for mass vaccination campaign workers in some sub-Saharan African countries and have revealed that it is associated with faster, completed payments and motivated health workers. To our knowledge, no such studies have been done in the Ugandan context.

**WHAT THIS STUDY ADDS**

- The study provides an account of the process for a partner-implemented digital payment system for paying mass vaccination campaign health workers in the Uganda context. It highlights that the partner-implemented digital payment was perceived to lead to more timely and complete payments to health workers and was thus perceived as feasible in the Ugandan context. The success of the partner-implemented digital systems was linked to the use of a dedicated digital payment team that ensured the timely capture, verification and approval of beneficiary payment details at different levels. The study also revealed that district officials felt that collaborating with development partners to conduct the payment freed up their time, allowing them to dedicate more time to monitoring and managing the delivery of the Campaign.

**HOW THIS STUDY MIGHT AFFECT RESEARCH, PRACTICE OR POLICY**

- This study’s findings (1) suggest that the government can partner with development partners to support the payment of mass vaccination campaign workers, and this has potential to improve both the efficiency and effectiveness of the campaign delivery and payment of campaign workers if adopted and (2) support the automation of the verification processes given the manual nature of current verification processes.

## Introduction

 Yellow fever, a viral infectious disease, remains a significant global public health threat. As an endemic country, Uganda continues to experience periodic outbreaks of yellow fever,^1^ highlighting the need for comprehensive vaccination campaigns. Mass immunisation efforts have proven effective in reducing the incidence and mortality of yellow fever,^2^ with numerous African nations successfully implementing campaigns, supported by the Global Alliance for Vaccines and Immunisation and the WHO.^3 4^ These efforts often draw on the practical knowledge and best practices gained from implementing large-scale government-led polio mass vaccination initiatives.^5^

Timely and complete payment of vaccination health workers is crucial for the successful implementation of such mass campaigns. However, persistent challenges with traditional cash-based payment systems have led to delayed campaigns, poor-quality operations and a demotivated workforce.^5 6^ Consequently, digital payments, particularly mobile money payment systems, have been embraced by government and non-government institutions to enhance efficiency and ensure quicker, more secure disbursements, thereby fostering satisfaction and morale.^7^ Historically, mass immunisation campaign payments, including payments to beneficiaries such as front-line health workers, have been managed by district local governments through cash or digital (mobile money) based systems. However, these have faced quality issues, including payment delays and non-payment to some beneficiaries.^8^

Some digital payment initiatives have shown promise in other contexts in response to payment challenges. Pilot programmes in several African region countries have demonstrated potential for timely and complete payments to healthcare workers, thereby contributing significantly to the success of vaccination campaigns.^8 9^ For example, nearly all campaign health workers in pilot countries received a timely and complete payment through their mobile phones, showcasing the potential of the digitised payment mechanism.^8^ Similarly, these initiatives have demonstrated the added value of engaging private partners to disburse and account for the immunisation activity funds directly, and this ensured transparency and effectiveness.^8 9^

In Uganda, despite accelerated digitisation efforts following the COVID-19 pandemic, the government’s primary digital payment platform faced significant issues, including delays, incomplete or non-payment, which hindered the efficient compensation of health workers in previous national immunisation campaigns. These payment challenges were largely due to inaccurate beneficiary details and the cumbersome nature of manual data capture, verification and upload processes, which hindered the timely and complete compensation of campaign workers.^10^,^12^

Payment systems in previous national immunisation campaigns also did not involve intermediaries since the district local governments made payments directly to the beneficiaries. However, due to accountability concerns, the Ministry of Health (MoH) contracted implementing partners (IPs), which were non-governmental organisations or autonomous institutions with established payment infrastructures, for the first phase of the yellow fever mass vaccination campaign in 2023 to disburse funds to campaign workers digitally. The MoH expected that payment through the IPs would result in more efficient and effective management of financial resources for the campaign. The IPs were responsible for managing financial resources, processing payments to vaccination workers and ensuring accountability for funds within their designated regions.

Considering that previous national immunisation campaigns primarily relied on government payment systems, there was a limited understanding of the implementation process and feasibility, particularly regarding the timeliness and completeness of partner-managed digital payments in vaccination campaigns in Uganda. This study, therefore, aimed to document the implementation process and examine stakeholder perceptions regarding the feasibility, particularly the timeliness and completeness of payments through a partner-managed digital payment system for campaign health workers.

## Methods

### Study design

This qualitative study was conducted between January and March 2025. The study used key informant interviews (KIIs) and in-depth interviews (IDIs) to document the experiences of stakeholders who participated in the planning and execution of the first phase of the mass vaccination campaign against yellow fever in Uganda.

### Study setting, context and population

Uganda, a home to nearly 46 million residents, is a country where yellow fever is endemic.^13^ The MoH in Uganda, with support from Gavi, the Vaccine Alliance, WHO, the US Centers for Disease Control and Prevention and other funders and development partners implemented a mass yellow fever vaccination campaign in a phased approach. The vaccination campaign was carried out between June 2023 and October 2024.^13^ According to the previous unpublished report from the MoH, the campaign’s first phase was conducted in June 2023. It covered 51 districts in 6 subregions of Lango, Acholi, West Nile, Rwenzori, Bunyoro and Kigezi, targeting all persons aged 9 months to 60 years, totalling 13 363 706. (MoH, unpublished report).

In each of the six subregions, the MoH assigned one IP to specifically plan and implement digital payments to all individuals who supported the vaccination campaign. The IPs per region included the Joint Clinical Research Center (JCRC), which is an autonomous Medical Research Institute in Uganda. JCRC supported the Lango and Kigezi subregions. The AIDS Support Organisation (TASO) Uganda, an indigenous HIV/AIDS service non-governmental organisation in Uganda, supported the Acholi subregion. The Infectious Diseases Institute (IDI), established within Makerere University, is a Ugandan not-for-profit organisation that aims to strengthen health systems in Africa, with a strong emphasis on infectious diseases. IDI supported the West Nile subregion. The Baylor College of Medicine Children’s Foundation-Uganda (Baylor Foundation Uganda) is an indigenous, not-for-profit organisation that aims to provide high-quality family-centred healthcare, education and research to reduce morbidity and mortality. Baylor Foundation Uganda supported the Rwenzori and Bunyoro subregions.

The study was conducted in four districts (Lango, Hoima, Amuuru and Kikuube) situated within three of the six subregions (Lango, Amuru and Bunyoro). The study population comprised individuals who participated in the planning and implementation of the yellow fever immunisation mass campaign. They included stakeholders, vaccination campaign workers, that is, vaccinators and mobilisers, district leaders, MoH officials, particularly the staff of the expanded programme on immunisation and digital payment IPs.

### Participants and selection criteria 

The study participants were selected using purposive and random sampling. From the six subregions, including West Nile, Acholi, Bunyoro, Kigezi, Toro and Lango, that the first phase of the yellow fever mass vaccination campaign covered, we randomly selected three regions, namely Lango, Acholi and Bunyoro (specifically, Hoima), to be included in the study. Thereafter, we randomly selected and included four districts, including Lira (from Lango subregion), Amuru (from Acholi subregion), Hoima and Kikuube districts (from Bunyoro subregion). A random selection of regions and districts was conducted to minimise bias and enhance geographic diversity.

#### Key informant participants selection

We selected and interviewed the following categories of participants:

1. The MoH expanded programme on immunisation staff (n=4) who were selected because of their direct and substantial involvement in the campaign’s planning and execution at the national level.
2. Key informants from IPs’ national (head) offices (n=3) and district level offices (n=4). The organisations included Baylor Uganda (for Hoima and Kikuube districts), TASO Uganda (for Amuru district), and JCRC (for Lira district). Supervisors at these organisations recommended these representatives/staff for participation in the study because of their substantial involvement in planning and implementing the digital payment at the national and district levels.
3. At the district local government level for each of the four study districts, we selected district officials, including the District Health Officer (DHO), Assistant DHOs, Chief Finance Officer and Chief Administrative Officer or their representatives/assistants (n=14). They were selected because of the positions they hold and their direct involvement in managing the yellow fever vaccination campaign in their respective districts.

### IDI participant selection

In addition, we selected three vaccination campaign frontline health workers from each of the four districts. These included two vaccinators and one mobiliser, n=12. Those selected were recommended by their respective supervisors at the district level based on their participation in the yellow fever campaign implementation.

Saturation was achieved after 37 key informant and IDIs. At this point, no new information was noted, and subsequently, data collection was stopped. See table 1 for a summary of study participants.

**Table 1:** Summary of interviewed study participants by category and location

Participant category	National level	District				Total interviewed
	UNEPI/National	Amuru	Lira	Hoima	Kikuube	
Ministry of health officials	4					4
Digital payment implementing partners (national level/head office)	3					3
Digital payment implementing partners (district/regional)		1	1	1	1	4
District local government stakeholders/leaders		4	4	3	3	14
Health workers (Vaccinators)		2	2	2	2	8
Health workers (Mobilisers like village health team members)		1	1	1	1	4
Total	7	8	8	7	7	37

UNEPI, Uganda National Expanded Programme on Immunization.

### Data collection tools and procedures

The study used semistructured IDI and KII interview guides to collect data from in-depth and KII participants. The IDI and KII guides were used to elicit information on key topics related to digital (mobile money) payments of health workers (vaccinators and mobilisers) who supported the recently concluded first phase of mass vaccination against yellow fever in Uganda. The researchers, some of whom are experts in this field, developed the KII and IDI guides.

We recruited and trained six research assistants, who were fluent in English and possessed at least a bachelor’s degree and had extensive experience conducting KIIs. The research assistants interviewed the district stakeholders and health workers, while two research investigators, including ME and CO, interviewed national-level participants.

We conducted audio-recorded face-to-face interviews in English with each participant. Interviews took place at the workplaces of participants, except for mobilisers, who were mainly interviewed at their respective homes. Additionally, during the interview process, the interviewers also captured key or emerging points. All the participants selected and interviewed were fluent in English, and therefore, there was no need to translate the interview guides into the local language. On average, the IDIs took about 60 min each, while each KII lasted around 75 min. All data were collected between January and March 2024.

### Data management and analysis

Audio recordings were transcribed verbatim, removing any identifiable information about participants, and entered into ATLAS.ti V.8.0. We analysed all the data using the thematic analysis technique. The transcripts were coded according to the coding framework initially reviewed to identify themes and subthemes and develop a coding framework. One of the investigators, together with an experienced research assistant, independently coded the transcripts using a coding framework. These were later compared, and any coding disagreements were resolved through discussion. The coding framework was revised periodically to incorporate new, emerging codes. Each statement was read and assigned a code based on its keywords. The main areas of our exploration included the partner-implemented digital payment process (implementation), the feasibility of digital payment (ie, timeliness), the likelihood of receiving all expected funds, as well as the perceived effect of partner-implemented digital payment on the motivation, satisfaction and performance of vaccination campaign health workers. We grouped the codes into categories and then identified emerging themes following the principles of thematic analysis.^14 15^ In presenting results, themes and subthemes were illustrated with representative quotes.

## Results

### Partner-implemented digital payment process

According to the MoH directive, the IPs paid all participants in the yellow fever mass vaccination campaign digitally. The payment process comprised key components, including preparation to conduct digital payment, capture, verification, approval of beneficiaries’ digital payment details, resolving complaints and execution of payments.

### Preparation to conduct digital payments

#### Several activities were undertaken to prepare for digital payment

The MoH assessed IPs’ existing digital payment systems and found them robust and aligned with its (the MoH’s) financial guidelines before deployment. Partners primarily integrated standard MoH forms, such as attendance sheets, vehicle logbooks and report templates, into their pre-existing mobile money payment processes. This minimal need for system modification led respondents to perceive partner-implemented digital payment systems as highly feasible due to their alignment with government protocols. As a key informant from IP Baylor College Uganda noted, “We used our Baylor systems… The only change was to use the Government of Uganda forms, but the process remained the same. So, we onboarded the Government of Uganda forms within our system…”. In addition, the results showed that during the training on yellow fever mass vaccination of the identified participants (vaccination health workers), organisers of the vaccination campaign clearly explained to the health workers that all payments would be made digitally through mobile money 1 week after the work was completed.

Furthermore, in consultation with the districts, IPs identified and trained some district officials as dedicated digital payment system records officials/assistants. These officials received training to accurately capture beneficiaries’ details and individual payment information using registration and digital payment forms. They were integrated into and became members of the vaccination teams. Regarding the training and guidelines for the required participants’ payment details, a key informant from JCRC highlighted “We also had to do training for the district officials… because electronic payments have criteria which you follow, ie, we needed phone numbers which are in somebody’s names, and if it’s not in their names, then they should put a consent form…”. Respondents felt this approach to training and data collection was important for improving the quality of the data and minimising errors related to incorrect beneficiary details and ensuring timely and accurate payment.

### Capture, verification and approval of beneficiaries’ digital payment details, resolving complaints and executing payments

Partners employed a largely consistent, multi-tiered system (eg, on-site, district, regional and national) for verifying and approving payments, as well as resolving payment complaints. Therefore, beneficiaries’ payment data moved through a clear, sequential process involving verification at each of the various levels to ensure quality and thorough accountability. Specifically, data quality checks to support the payment approval process started at the vaccination sites, where the digital payment records assistants captured and verified the digital payment details (eg, attendance and mobile money records) of the beneficiaries. According to interviewed participants, the emphasis on data quality at vaccination sites, particularly data capture and verification by dedicated records assistants at the vaccination sites, was a critical quality assurance starting point for the entire payment process. A key informant from TASO Uganda stated, “…. I think we needed to have our structures play that role of ensuring that the documents at the vaccination site are collected on that same day, unlike thinking that the districts would play that role”. At the district level, the IP officials also worked closely with the DHOs to further verify and approve the submitted payment details. At the national level, partners had dedicated verification teams that further reviewed the huge volume of payment documents sent from the districts and the regions. These review teams also constituted another data quality layer that checked payment data for consistency in a timelier manner. A key informant from Baylor College Uganda explained, “In Baylor, our cluster down, there was the first layer of review of accountability. About three districts report to a cluster. Now, at that cluster, there was a focal person who was assigned the role of verifying accountabilities in line with the financing guidelines. At that level, that person then submits to the regional finance person, who does a final review of that accountability and then he is able to authorise and subsequently it goes through the electronic layers of approval, then finance and then another programmes person also approves. Payment was effected electronically”.

Once the verification was completed and all beneficiary details in a batch of participant forms matched, the IPs effected payment for all participants in that particular batch using their respective existing mobile money payment systems. A key informant from Baylor College Uganda clarified “…we used our normal mobile money payment systems to pay off implemented activities that had their accountability records submitted and had been verified at that level for payment, and of course, the verification also followed the Baylor process.”

Regarding resolving payment complaints, the study results further showed that despite efforts and procedures to verify participants’ details before effecting payments, some complaints for non and/or delayed payment and or payment of the wrong persons still arose. The results further revealed that the IPs, working with the districts, put or used the same sequential processes and levels for investigating and resolving the complaints in a timely manner. A key informant from Baylor College Uganda further explained “So, complaints came in; including missed payments. Surprisingly because there was feedback mechanism ….our teams would generate proof of payment, share with districts in resolving those complaints. This is now the strength of mobile money platforms”. Overall, the results showed that some of such instances stemmed from health workers providing unmatched personal details like mobile money numbers registered in their respective spouses’ names without declaring it. This was contrary to the requirement that these should be matched, and if the spouse’s phone number is used, then it should be declared and consent provided for the funds to be deposited or sent to such phone numbers.

Thus, the collaborative nature in which these different levels worked, and also with the district health offices, and the fact that the verification teams mostly worked simultaneously during implementation, was considered an important factor. All these verification layers and the collaborative working approach contributed to timelier and complete payments to the beneficiaries. A key informant from TASO Uganda elaborated, “…as a key lesson, collecting data during the same time as implementation shortens the period and increases the accuracy of payment systems. One you do it at the same time when the people who are vaccinating are there so it is easy to verify, and then mistakes are easy to correct, and they take a shorter period of time and in the end the payments will be faster for those that have carried out the vaccination…”.

### Perceived benefits to the district local governments and MoH of partner-implemented digital payment

#### Partner-implemented digital payment improved coordination

District officials reported that the partner-led system more effectively managed payment processes and accountability, allowing them to focus on implementing the programmatic components of the campaign. A MoH key informant noted, “it was meant to relieve the districts of the burden of financial management and the delays and processes that are usually in our system as government, so it was meant to ease considering the limited time and the accountability challenge that had been there that time given that the districts had accumulated amount of funds from previous campaigns to account to the MoH”. It was suggested that while some district officials initially perceived this as a challenge to their mandate, they later acknowledged its benefits, as a key informant from Baylor College Uganda observed “Turned out to them as beneficial. At first, they said you are taking our mandate, but later they said that the way you are working; you are actually more transparent, and I think this is going to benefit us, but also you are taking away the burden of accounting for resources from us.”

At the national level, MoH respondents found the coordination of the mass vaccination exercise to be much simpler, as dealing with a few IPs, rather than numerous individual districts, was easier. The approach also facilitated timely payments and submission of accountabilities (financial records) to the MoH. An MOH official and key informant highlighted this advantage, “What worked well for us is that we no longer have any accountabilities with many administrative units. We know that when you have accountabilities pending from 100 districts, it is different from asking it from four organizations. So, it eases accountability for us.”

#### Improved payment timeliness and completeness

Participants reported that the partner-implemented digital payment improved the timeliness and completeness of the payment, indicating that it was not only feasible to pay health workers using the partner-led payment system but also ensure the beneficiaries receive timely and complete payments. Regarding the timeliness of payment, a key informant from JCRC stated “Some (payments) took two days, which was very good, some took one week”.

However, it is important to note that timeliness was only possible if the IPs (officials) received all the required payment details’ documentation in time from the records assistants who also received it from the front-line health workers. Second, the documentation had to be accurate. Therefore, in some cases, payments were delayed for a period exceeding a week, or even a month, because of inaccuracies in the documentation or delayed submission. A key informant from Baylor College, Uganda, expounded, “So, on average that was prolonged to about 7–8 days even 10 roughly. ….I think in all ways I did not see anything going beyond 10 days apart from the usual issues of missing paperwork. That is what delayed. Mismatches between the mobile number and the names that are registered. Those delayed the process so it kind of doubled our turnaround time.”

#### Perceived likelihood of receiving a complete payment

Health workers reported that most of them, and in some instances, all members of some groups, were paid and received their full amount, and thus, they were motivated to continue working and derived satisfaction from it. When asked if they believed that with a partner-implemented digital payment system, they would likely receive all the funds they worked for, one IDI participant, a vaccinator from Kikuube district, stated and explained that “Yes, of course. Just like I have been saying, e-cash reduces the number of people who get hold of the funds, and you never know there are consequences; when it is paid directly from the implementing partner, you get the exact amount that is planned and worked for, and if it is 5 days, then you get 5 days for you”.

In another IDI interview, a vaccination mobiliser from Hoima district also reinforced this by saying “….we were assured that we shall receive our money in full and in time from wherever you are. You could be there in your garden and see a message coming on the phone; you go and withdraw your money, and it helps you. It is, therefore, a very, very good method”. Indeed, the results showed that the majority of the beneficiaries received their payment as planned, as a key informant from Baylor College Uganda indicated that they made complete payments to the majority of beneficiaries. “For the health workers in my region, we paid 99%”.

### Perceived effect of partner-implemented digital payment on the motivation and satisfaction of vaccination campaign health workers

In further discussions, the health workers emphasised that the partner-implemented digital payment system was more motivating and satisfying compared with the government-led system. This was largely attributed to the timeliness and completeness of the partner-led payment system. One IDI participant who was a mobiliser in Hoima district explained, “It makes us very happy because you get your money on time. Even when you come for that exercise, they will tell you that you are going to get all your money on time. Knowing money will come at once is very encouraging. You work well knowing your money is coming on time and intact”. One national level key informant from TASO further clarified, “They were paid money faster than they had been paid before in the different campaigns”.

Interviews with recipients of the payments reiterated that there were some cases of delayed payments, and these delays negatively impacted the motivation of vaccination health workers. In a further probe into this, participants disclosed that delayed payments did not impact the motivation of different health workers equally. Rather, it had a greater negative impact on the motivation of Village Health Teams (VHTs) and mobilisers than on formal or professional health workers, such as vaccinators. One key informant from Lira district explained, “…of course, now the motivation goes down, but for us as health workers, that is our career, and we are used to it. However, the problem arises with community leaders, VHTs and Local Council 1, as well as any other individuals you may have sourced from outside…”. In an IDI interview, one vaccinator from Lira district added to this by saying, “Ok, for me, as a health worker, it does not affect my motivation to perform because immunization is part of my work, and the government is paying me for that. Actually, we are supposed to do immunizations”.

## Discussion

This qualitative study examined the partner-implemented digital payment system in the first phase of the yellow fever mass vaccination campaign for health workers in Uganda. The findings thus provide insight into the feasibility, specifically in terms of timeliness and completeness of a digital payment system when it is partner-implemented. Our study revealed that overall, because of the timeliness and completeness, most participants from all categories and levels preferred and recommended a partner-implemented digital payment system mainly because of the positive experiences they got from it. This is in keeping with other studies, which have shown that most vaccinators preferred to be paid using mobile money and that the vaccinators’ positive payment experience was linked to the timeliness of payments.^8 16^

Previous and similar studies indicated several factors for timely, complete payments to frontline health workers participating in mass immunisation campaigns and ultimately a positive experience of such beneficiaries. Among them was the systematic engagement of IPs and government counterparts at various levels, which helped to provide checks and balances throughout the funds management process. This included administrative activities such as data entry, recording worker attendance and overseeing payments, all of which contributed to the credibility and transparency of the digital payment systems as well as the timeliness and completeness of payments.^8 9^ Similarly, our study showed that several factors contributed to the improved timeliness and completeness observed in the partner-implemented digital payment system. The IPs had dedicated teams at various levels, including vaccination sites, district, cluster, regional and national levels, to verify and process payment records accurately and in a timely manner. This thorough review process was thus a critical data quality assurance measure. It reduced the inaccuracies that often lead to delayed payments and ensured complete payments to the majority of beneficiaries. In addition, partners adopted and used pre-existing, robust and tested digital/mobile money payment systems, which were also flexible enough to allow them to add other components from the Government system.

These findings imply the potential value of using partner-implemented digital payment systems to promote timely and complete payment of campaign health workers. The MoH should, therefore, consider such partnerships to promote the efficient payment of campaign workers during mass immunisation campaigns. Furthermore, the district stakeholders interviewed felt that the partner-implementation of the payment system freed up the time of the district implementers, allowing them to concentrate on the programmatic issues related to the campaign. Such trade-offs may contribute to improving the quality of immunisation and other national health campaigns.

Most of the digital payment processes were manual, and as we have shown, the process could take between two and 10 days, but also up to a month, particularly in some cases where documentation was lacking, inadequate or inaccurate. Work elsewhere has shown that digital payments can be done in less than a week.^5^ In particular, a similar initiative in Côte d’Ivoire recorded an impressive average digital payment time of 2 hours.^5^ Payment delays can be mitigated through the automation of various payment processes, such as data capture, review and approval of payment documentation, thereby reducing payment time. Additionally, it will require further investment to equip the different payment centres with electronic financial systems. More research about the cost implications of paying digitally is therefore warranted.

Our paper also highlights the importance of communication between the beneficiaries and the payers. Health workers were informed that the payment would be made within a week. This set an expectation from the payment beneficiaries, and when it was indeed met (payments made within a week), it was satisfying and motivating. Timely communication of payment periods and delays in payment can help reduce dissatisfaction with mobile payment. Existing digital payment evidence reveals that delay in payment was one of the top two reasons for the unsatisfactory experience and health worker demotivation, particularly in Côte d'Ivoire.^5 16^ This suggests that to ensure frontline health workers’ satisfaction and motivation from the digital payment system, it is not only important to ensure timely payments but also to communicate what to expect.

### Study strengths and limitations

One strength of this study was the inclusion of a diverse team of participants that included frontline health workers such as vaccinators and mobilisers, digital payment IP representatives, as well as district and national-level participants. The concordance from the data obtained from these different participants reinforced the validity of our results. In addition, many of the issues presented here also arose in similar qualitative studies designed to investigate similar perspectives of stakeholders regarding the payment of frontline health workers for mass vaccination in other similar settings. Furthermore, this study triangulated data collection methods, including in-depth and KIIs, to explore the study themes. Triangulation has been widely adopted in qualitative research to investigate the validity of the data and their conclusions.^14^

The potential limitation was that the study did not specifically seek to quantify or collect data on the proportion of vaccination campaign health workers who got paid and on time. Such data, if captured, would have added weight to the findings that the partner-implemented digital payment increased the likelihood of being paid promptly. Several participants were also recommended by their supervisors. While this was necessary to identify individuals with specific experience in implementation, it may have introduced some selection bias. We believe our approach to triangulating the findings with additional perspectives helped to mitigate this potential bias.

## Conclusions

A well-coordinated digital payment process has the potential to enhance vaccination campaign worker morale, satisfaction and performance, which are critical to campaign success. This study found that partner-led digital payment had positive benefits, including more complete and timely payments, simpler coordination and fewer accountability challenges, signalling improvements in the efficiency and reliability of the payment system. However, the effective execution of digital payment systems requires deliberate investment in their administration. In particular, human resources were essential for the accurate preparation, capture and verification of beneficiary payment details within the payment platform. Establishing dedicated digital payment teams at various levels can help ensure timely and complete payments. Manual systems, however, tend to be resource-intensive and are still prone to human error. Automation should therefore be considered. As noted in the limitations section, this study was based on qualitative data, which may affect the generalisability of findings. We, therefore, recommend further, more rigorous evaluations of partner-led digital payment systems to guide potential scale-up and national adoption.

This study suggests that the first phase of the yellow fever mass vaccination campaign demonstrated that partner implementation of digital payment improved (digital payment) in terms of improved timeliness and complete (total amount worked for) payment. Thus, it is potentially feasible and could potentially improve campaign effectiveness since other stakeholders and local government officials dedicate their attention to the programme aspect of the campaign. The MoH should consider a partner-implemented digital payment system as a formidable alternative to the primary, that is, local government or district-implemented payment system. However, for digital payments to work well, the IPs and the MoH should ensure dedicated administrative investments, including setting up dedicated digital payment teams to ensure the timely capture and verification of beneficiary payment details at different levels to enable timely and complete payment. We recommend further and more rigorous assessment of partner-implemented digital payment systems to inform the potential rollout or adoption.

## Data Availability

Data are available on reasonable request.
